# The Association of Socio-Economic Factors and Indigenous Crops on the Food Security Status of Farming Households in KwaZulu-Natal Province

**DOI:** 10.3390/agriculture14030415

**Published:** 2024-03-04

**Authors:** Nomfundo Shelembe, Simphiwe Innocentia Hlatshwayo, Albert Modi, Tafadzwanashe Mabhaudhi, Mjabuliseni Simon Cloapas Ngidi

**Affiliations:** 1 Centre for Transformative Agricultural and Food Systems, School of Agricultural, Earth and Environmental Sciences, College of Agriculture, Engineering and Science, University of KwaZulu-Natal, Private Bag X01, Scottsville, Pietermaritzburg 3201, South Africa; 2 African Centre for Food Security, School of Agricultural, Earth and Environmental Sciences, College of Agriculture, Engineering and Science, University of KwaZulu-Natal, Private Bag X01, Scottsville, Pietermaritzburg 3201, South Africa; 3 Centre on Climate Change and Planetary Health, London School of Hygiene and Tropical Medicine, Keppel Street, London WC1E 7HT, UK; 4 Department of Agricultural Extension and Rural Resource Management, School of Agricultural, Earth and Environmental Sciences, College of Agriculture, Engineering and Science, University of KwaZulu-Natal, Private Bag X01, Scottsville, Pietermaritzburg 3201, South Africa

**Keywords:** extended ordered probit model, food security status, indigenous crops, household food insecurity access scale

## Abstract

Indigenous crops have been proposed as part of a solution for household food security and sustainable farming systems. However, they have been overlooked and underutilised by households and farmers despite their potential contribution to household food security. The objective of this paper was to determine the association of socio-economic factors and indigenous crops with the household food security of farming households. About 260 farming households were selected using a simple random sampling procedure. The food security status was measured through the use of the Household Food Insecurity Access Scale (HFIAS). The Chi-square test and extended ordered probit regression model assessed the relationship of socio-economic factors and indigenous crops with household food security status. The results from the HFIAS showed that farming households were largely in the mildly and moderately food-insecure categories, with 34.2% and 36.2% of the sampled farmers found in these categories, respectively. The Chi-square test showed a statistically significant relationship between food security status and socio-economic factors. Young men and everyone were perceived to be the ones likely to consume indigenous crops. Consumption of indigenous crops was perceived to be associated with food security. The results also showed that farming experience is likely to positively contribute to the food security status of the farming households. Selling indigenous crops in a formal market is perceived to be associated with food security compared to selling in an informal market. This study concludes that consuming indigenous crops is likely associated with improved food security. Identifying an appropriate market for sales of indigenous crops is imperative. Government, extension officers, and nutritionists must conduct training workshops to encourage households to grow, market and buy indigenous crops. Government and policymakers need to include indigenous crops in the national food and nutrition security policy and create formal markets for indigenous crops.

## 1 Introduction

In South Africa, the agriculture system is a crucial industry that contributes significantly to alleviating socio-economic problems by fostering development and food security [1–3]. It is vital in economic growth, employment creation, poverty alleviation, and food and nutrition security [4–7]. It is characterised by commercial agriculture, which utilises 87% of the agricultural land, with only 13% scattered among emerging and subsistence agriculture [8]. About 8.5 million individuals in South Africa depend on agriculture for employment and income, with the sector contributing to about 3% of the Gross Domestic Product (GDP) and 7% of formal employment [9]. Similarly, agriculture is the main source of work and income in many parts of Southern Africa [10]. The large increase in the South African population through the years places great pressure on the country’s food production and the need to provide more nutritious food for vulnerable communities [11]. Thus, increasing agricultural productivity depends on effective measures to reduce poverty and hunger, particularly in vulnerable groups.

As rural and peri-urban area residents continue to be vulnerable, farming has been key to the survival of households. It contributes significantly to households’ caloric intake, ranging from 42% to 58% [12]. Subsistence and smallholder agriculture further contribute to improving dietary diversity and food security, reducing the need for overreliance on purchased food [13]. Poor households in South Africa generally lack the purchasing power to buy food from the markets [14]. Therefore, involvement in indigenous crop production is seen as an alternative towards making food available and accessible for these households. Indigenous crops are an alternative that is less costly for farming households to produce. Indigenous crops are a plausible pathway towards attaining the 2030 Sustainable Development Goals (SDGs) of no poverty, zero hunger, and gender equality [15]. These crops have been proposed due to their climate-resilient nature and adaptability among several environments [16,17]. While these crops have been labelled as an integral part of household food security [18], they are considered poor man’s food among individuals [19]. Some common indigenous crops in South Africa include sorghum, amaranthus, and sweet potatoes [20]. Indigenous crops can generally help cropping systems to become more sustainable whilst aiding the rise in population to ensure long-term food supply while minimising adverse effects on land, water, and air [21,22].

Despite the advantages of these crops, there are also several challenges that farming households face when cultivating indigenous crops. These challenges include the unavailability of seed and cultivating material, lack of market infrastructure, and competition from exotic crops such as maize, spinach, and cabbage [23]. Individuals from rural and urban areas generally cultivate and purchase exotic crops rather than indigenous crops as they are perceived to be more nutritious and tastier. This preference for exotic crops decreases indigenous knowledge transfer from older to younger generations and deprives people of consuming less costly nutritious foods [24]. Other challenges include genetic erosion, vulnerability to pests and diseases, and unavailability of varieties. This shows the need for breeding programmes to introduce new genotypes for improved traits and innovative technology to improve their production and adoption [25]. The challenges also include limited agricultural production, a lack of resources, inadequate transportation infrastructure, poor yield and quality, lack of market access, and lack of agricultural credit and land [26,27].

To fully reap the benefits of indigenous crops and address the SDGs relating to food and nutrition security, it is important to include female- and youth-headed farming house-holds. Female-headed house-holds participating in farming are more food insecure than male-headed households. Although males and females have limited agricultural resources, female-headed households are more limited due to cultural, traditional, and socio-economic factors [28]. This creates the need for targeted programmes to empower female farmers to reduce their vulnerability to food insecurity [29]. Moreover, this underlines the continued need for extensive support in production and marketing information for female-headed households to improve household food security [30–34].

Additionally, several papers list these crops as having low consumption rates, being underutilised, not being fully documented, and having limited production information [35–39]. This limits the growth and variety of production, consumption, agro-processing, and strengthening of the market value chain for indigenous crops. Farming households in South Africa are further characterised by unstable food availability (through their production with limited resources) and access (through limited income and high rates of unemployment), thus requiring more definitive and detailed information on producing these crops at a better scale to supply household needs and incur revenue.

Thus, it is important to explore whether farming households’ perspectives on these crops’ marketing potential, access, and marketing avenues affect their overall household food security status. Several papers have investigated how the awareness and perception of indigenous crops on individuals affect household food security in South Africa [34,37,39,40]. Mabhaudhi et al. [37] pointed out that a gap exists in mainstreaming indigenous crops in South Africa because there is a lack of broad knowledge of these crops, negative perceptions from farmers, and reliance on exotic crops across the country. Similarly, Modi [39] found that subsistence farmers of KwaZulu-Natal (KZN) had limited knowledge of indigenous crops and did not attach much importance to them. However, the study by Modi [39] did not focus on the association of indigenous crops with household food security. On the other hand, Zondi et al. [34] found that socio-economic factors affected the food security status of several households producing indigenous crops. However, the focus was on the Limpopo and Mpumalanga provinces of South Africa. The studies did not focus on the KwaZulu-Natal province, which has many households involved in agriculture. There is limited information that links both the socio-economic factors and indigenous crops together with their potential to improve food security. In light of this background, this study is designed to provide insight into the link between socio-economic factors, indigenous crops, and household food security status.

## 2 Materials and Methods

### 2.1 Description of the Study Area

**Figure 1:**
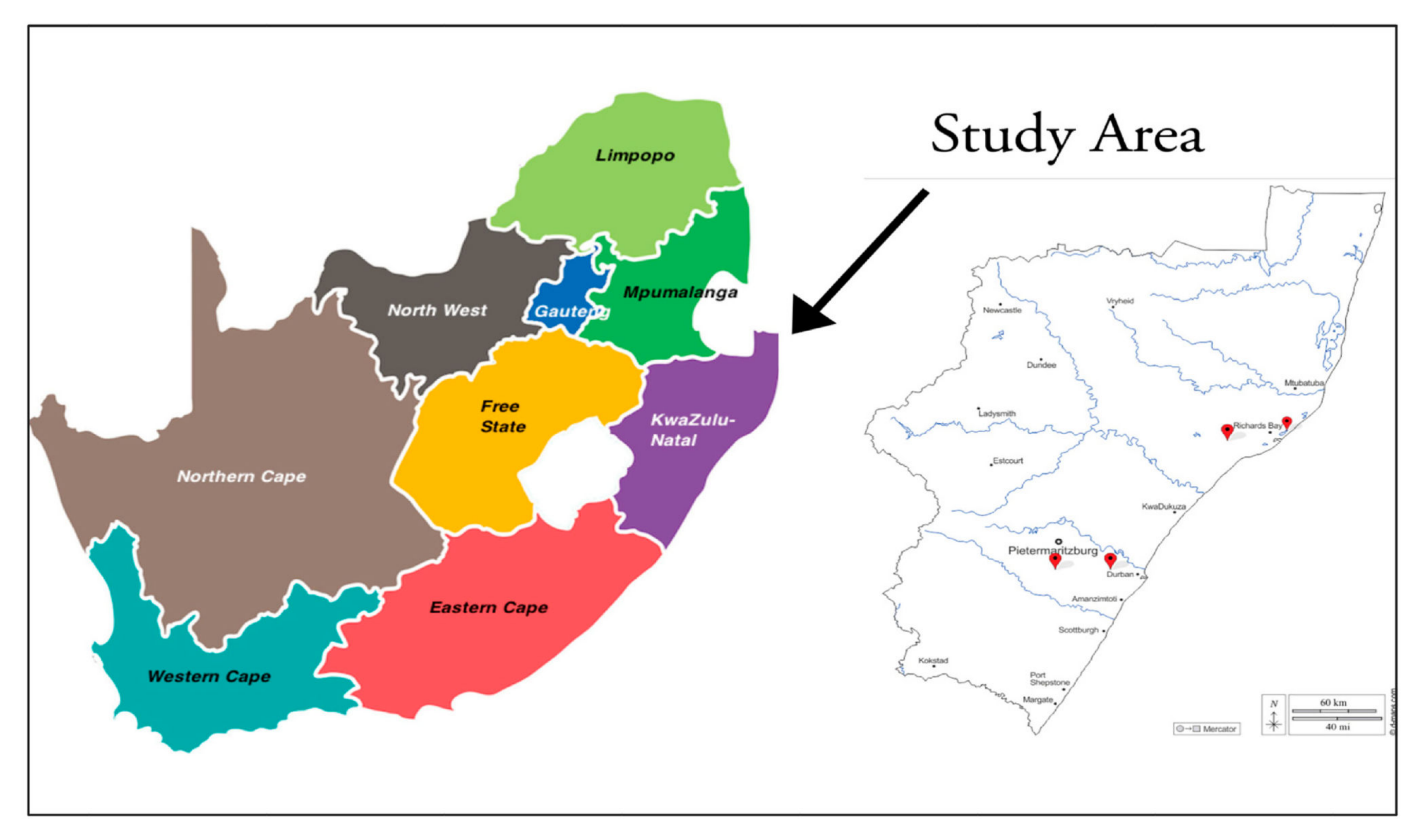
A map showing the KwaZulu-Natal study area [42].

This study was conducted in the KZN province of South Africa. A subtropical coastline, grasslands in the east, and a wide mountain range in the west characterise the province. It has a varied climate and diverse topography. The province experiences dry climates during June and high rainfall in January [41]. The coast is subtropical, with inland regions becoming progressively cold. The south coast has an estimated 1009 mm annual rainfall with an annual high temperature of 24.77 °C (76.59 °F) and a low temperature of 19.7 °C (67.46 °F). This study focused on three districts within KZN: uMgungundlovu, eThekwini, and Harry Gwala (Figure 1). From these three districts, five areas were selected for the study. These areas included Mbumbulu, Swayimane, Imbali, Shayamoya, and Cabazi locations. The study areas were chosen because they are part of a larger project focusing on commercialising indigenous crops where the University of KwaZulu-Natal partnered with the Technology Innovation Agency (TIA).

### 2.2 Data Collection

This study utilised quantitative methods. Data collection was conducted in 2023 using structured household questionnaires. Data collected included but were not limited to demographics and socio-economic characteristics, perceptions, food consumption, use of indigenous crops, naming, and how farmers value these crops economically. Zulu-speaking enumerators administered the questionnaire to the farmers. The farmers were selected from a sampling frame using simple random proportional sampling in the District Municipalities. A sampling frame is a unit of all individuals that contains the intended audience in research; it is used when the participants intended cannot be feasibly covered due to study limitations such as time and funding. This study utilised a list frame that contained different farmers of all ages, alphabetically listed from the chosen districts. The sample size was calculated using the 95% confidence interval and 5% margin of error based on the sampling frame of 567 farming households, with each household having an equal chance of being selected. This yielded a sample of 260 farming households. The uMgungundlovu district had a sample of 120 participants, Harry Gwala had 80 participants, and the eThekwini district had 60 participants.

### 2.3 Data Analysis

After completion of the fieldwork, questionnaires were checked for completeness, accuracy, and reusability. The Statistical Package for Social Sciences (IBM SPSS), version 28, and STATA, version 18, were used to compute descriptive statistics and quantitative models. To measure household food status, this study utilised HFIAS (please see Supplementary Material S1). The HFIAS measure examined three aspects of food insecurity: (1) feeling anxious and uncertain about the household’s food supply, (2) changing one’s diet’s quality, and (3) consuming less food overall. Nine questions made up the tool, which inquired about dietary or food consumption modifications households made due to having few resources to buy food in the 30 days before the survey interview date. The measurement followed a continuum that started with worry over food availability, then a decline in the quality and amount of food, and finally, going to bed hungry and without food all day and night. Based on the nine items, there were four progressively more severe levels of food insecurity. They were either in a state of food security or mild, moderate, or severe food insecurity.

### 2.4 Extended Ordered Probit Regression to Determine the Association between Indigenous Crop Factors and Household Food Security

An extended ordered l regression was used to investigate the association between indigenous crops and the household food security of the sampled households. The extended ordered probit model is suited for modelling with an ordered categorical dependent variable and accommodates issues with selection bias. To properly assess the factors affecting household food insecurity within the sample, this study used the ordered categories of the HFIAS (Q_1_ = 1; food secure: 0–1), Q_2_ = 2 (mildly food insecure: 2–8), Q_3_ = 3 (moderately food insecure: 9–17), and Q_4_ = 4 (severely food insecure: 18–27) as the dependent variable in the ordered probit regression [43–46]. The respective category for food security is unobserved and is denoted by the latent variable Qi*. The latent equation below models how Qi* differs with characteristics.

(1) $${\text{Q}}_{\text{i}}*={\text{X}}_{\text{i}}$$

The difference in the value that individual i derives from being food secure, slightly food secure, moderately food insecure, or severely food insecure is measured by Q_i_*; i is equal to 1, 2, 3, … n, where n is the total number of respondents. Every individual is a member of one of the four groups, and X is an exogenous variable vector. Hence, taking the value of 4 if the household was severely food insecure and 1 if a household was food secure, the implied probabilities are as follows [46]:

(2) $$\begin{array}{l} \text{Pr}\left\{{\text{Q}}_{\text{i}}=1|{\text{X}}_{\text{i}}\right\}=\Phi \left(-{\text{X}}_{\text{i}}\beta \right),\\ \text{Pr}\left\{{\text{Q}}_{\text{i}}=2|{\text{X}}_{\text{i}}\right\}=\Phi \left(\mu_{2}-{\text{X}}_{\text{i}}\beta \right)-\Phi \left(\mu -{\text{X}}_{\text{i}}\beta \right),\\ \text{Pr}\left\{{\text{Q}}_{\text{i}}=3|{\text{X}}_{\text{i}}\right\}=\Phi \left(\mu_{3}-{\text{X}}_{\text{i}}\beta \right)-\Phi \left(\mu_{2}-{\text{X}}_{\text{i}}\beta \right),\\ \text{Pr}\left\{{\text{Q}}_{\text{i}}=4|{\text{X}}_{\text{i}}\right\}=1-\Phi \left(\mu_{3}-{\text{X}}_{\text{i}}\beta \right). \end{array}$$

where μ_i_ is the parameter estimated jointly with β, and where the above probabilities enter the likelihood function, the maximum likelihood is used for estimation. The β coefficients are interpreted in terms of the equation’s underlying latent variable model. Thus, the probabilities of farming households falling between 1 and 4 can be written as follows;

(3) $$\text{Pr}\left({\text{Q}}_{\text{i}}=1\right)=\text{Φ}\left({\text{X}}_{\text{i}}\beta_{1}\right)$$

where Φ (·) is the cumulative distribution function of the standard normal. Table 1 below represents the dependent and independent variables used in the model. The independent variables were then hypothesized on their expected effect on the dependent variable.

#### 2.4.1 Justification for Proposed Variables

##### Indigenous Crop Access

Indigenous crop access refers to how farming households access indigenous crops. It is divided into three categories whereby farming households may either cultivate these crops in their home gardens, cultivate from other cultivated lands, or possibly collect from forests or wild velds where they naturally grow. Access to indigenous crops differs across different African countries. In South Africa, access to indigenous crops is limited to cultivation and collection in the wild, but these crops are not commonly accessed through formal markets such as supermarkets [35]. Contrastingly, in countries such as Kenya, indigenous crops are readily available in supermarkets [47]. Consistent access to indigenous crops in various ways has been linked to household food security [48]. Indigenous crop access has been hypothesised to positively influence household food security.

##### Indigenous Crop Consumption

Indigenous crop consumption refers to whether a farming household consumes or does not consume indigenous crops. Consumption of indigenous crops has been suggested to improve access to nutritional household food baskets for poor communities and vulnerable households [49]. The number of times a household consumes indigenous crops can contribute significantly to the diet of household members and, thus, their food security status. An increase in the consumption of indigenous crops is hypothesised to positively influence household food security.

##### Farming Period

The farming period refers to the years a farming household has participated in agriculture. This was divided into four categories: under 4 years, 4 to 10 years, 10 to 20 years, and greater than 20 years. Increasing the number of years a farming household participates in agriculture increases exposure to information and experience. The number of years a farming household has acquired in cultivating different crops in their home gardens exposes them to different practices, diversifying their diet and improving their yield [50]. This can lead to stable food security if utilised well [51]. An increase in the number of years of farming is hypothesised to positively influence household food security.

##### Indigenous Crops’ Perception

In this study, indigenous crops’ perception refers to which individuals farming house-holds perceive as those who should be consuming indigenous crops. This was divided into older women, older men, young women, young men, and everyone. In essence, all individuals should consume indigenous crops to gain their nutritional advantages. However, individuals’ negative perceptions of these crops have led to a decline in their consumption, ultimately linked to household food security [52]. According to [47], although indigenous crops are more nutritious than popular and commonly consumed exotic crops, negative attitudes constrain efforts to increase the consumption of indigenous crops [53]. This is further exacerbated by most exotic crops being easily accessible in markets whilst indigenous crops are rarely sold. Although various initiatives have changed consumers’ perceptions of indigenous crops, exotic crops are still far more popular [54]. Perceptions of indigenous crops are hypothesised to have a negative or positive influence on household food security.

##### Required Assistance

The required assistance variable refers to the production inputs that farming households listed as required to improve yield, income, and food purchasing power. These were divided into seeds, garden tools, fencing, shielding nets, and soil analysis services. Farming households receiving support from either government/research institutions through extension work have been linked to household food security [55–57]. This is hypothesised to have a positive influence on household food security.

##### Indigenous Crops’ Perceived Marketing Potential

This variable refers to whether farming households perceived indigenous crops as marketable. Considering that several studies have found the perception of indigenous crops to be negative [58–60], it is expected that farming households may find indigenous crops unmarketable. However, it is important to find out whether farmers negatively perceiving these crops will have any influence on their household food security status.

##### Indigenous Crops Are a Suitable Marketing Channel

This variable refers to the marketing channels farming households find suitable for indigenous crops. This was divided into four categories: local, formal, informal, or all markets. Indigenous crops are less popular in markets due to low levels of acceptability and access, limited market information, lack of processing technologies, and intrinsically weak value chains [34,35,61,62]. This limits the number of indigenous crop producers and may affect household food security in the long term. How farming households select marketing channels is hypothesised to negatively or positively influence household food security depending on the channel selected.

## 3 Results and Discussion

### 3.1 Socio-Economic Characteristics of the Sampled Farming Households

The survey was conducted in three districts within five different areas across the KwaZulu-Natal province in South Africa. Table 2 presents an overview of the demographic characteristics of the study participants. The descriptive results showed that more respondents were female than male, with 63.5% of farmers being female. These findings align with various studies [63–65] in South Africa, indicating that more women participate in household agriculture. This is unsurprising as government efforts to promote women in agriculture are being intensified [66]. The results also showed that farmers were scattered among the different age groups, with the highest number of farmers being older than 65 years (22.3%). This was followed by ages 35 to 44 (18.5%), and after that, a similar number for the ages of 25 to 34 years (16.5%) and 45 to 54 years with 16.9%. Farmers between the ages of 55 and 64 years had only 15.0%, and the ages of 18 to 24 years had only 10.8% of the sample.

More respondents were unmarried, with approximately 55.8% of the respondents found to be unmarried compared to 44.2% of the married respondents. Most household respondents (55.4%) had obtained secondary education, with very few attending post-high school education. Approximately 37.7% of the respondents were unemployed, and 32.7% reported being recipients of social grants. The unemployment levels were slightly higher than those in the rest of the country, where 28.8% of the population in South Africa is unemployed [67]. Most farming households had an income of ZAR 0–500 and ZAR 501–1000, with 18.5% of the households from each range reporting having received such an income per month.

### 3.2 Prevalence of Food (in)Security Amongst the Sampled Farming Households

#### Food (in)Security Situation amongst the Farming Households

The prevalence of food security was measured through the use of HFIAS. Table 3 shows the percentage of farming households that experienced food shortages 30 days before the survey. Most households (77.7%) indicated that they could not consume the foods they preferred because they lacked resources. This was followed by households who indicated that they had to eat some foods they did not want. In terms of frequency of occurrence, most households (31.2%) indicated that for more than 10 times in the past 30 days, prior to the survey, they could not eat the foods they preferred. These higher percentages indicate that farming households experience some levels of food insecurity.

**Figure 2:**
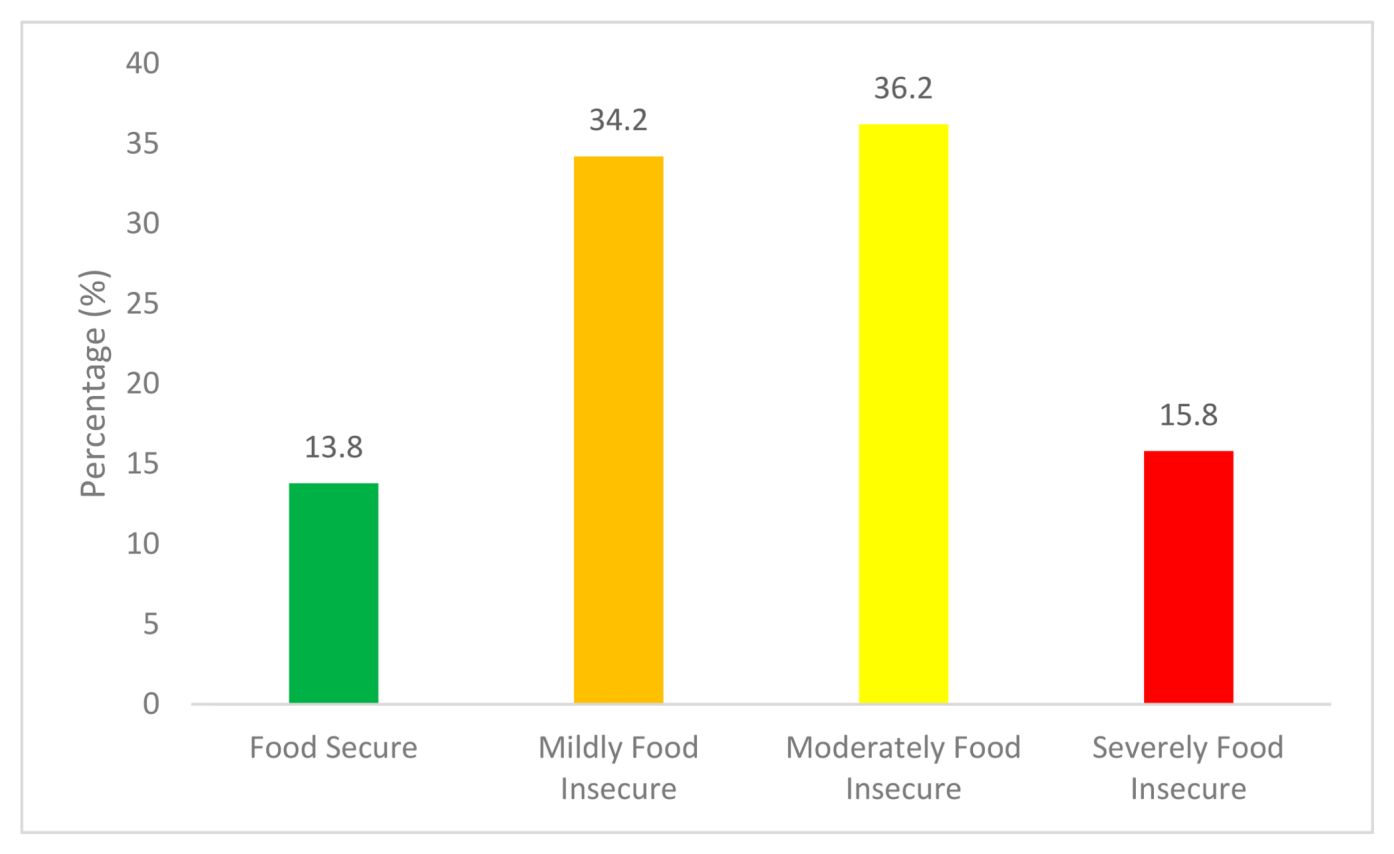
Household food insecurity occurrence.

The level of household food insecurity is shown in Figure 2 below. The analysis for food insecurity status indicates that 13.8% of the farming households were food secure, 34.2% were mildly food insecure, 36.2% were moderately food insecure, and 15.8% were severely food insecure. These results show that several farming households within the three districts experienced some difficulties in accessing a healthy and nutritious food basket. These results align with those from [68–71], who reported that households could not access and maintain a healthy food basket throughout the month due to unemployment and many household members needing food.

### 3.3 Factors Associated with Indigenous Crops and Their Contribution to Household Food Security

Factors affecting the food security status were analysed using the Chi-square test and the extended ordered probit model. The former analysed the effect of socio-economic parameters on household food security, while the latter model was used when analysing the effect of indigenous crops on household food security.

#### 3.3.1 Association between Socio-Economic Parameters and Household Food *(in)Security

Table 4 presents the association between socio-economic characteristics and food security. A statistically significant relationship exists between the food security status and gender, household monthly income, and number of household members (*p* < 0.01). The findings in Table 4 show more female farming households, and more female-headed farming households are food insecure than male-headed households. These results are not surprising because it is reported by various studies in South Africa and Africa in general that while more women are involved in smallholder agriculture, farm finances or income and associated decisions reside with males [72–74].

The results also show that the number of household members is significantly associated with household food insecurity. This means that the higher the number of household members, the more the household experiences food needs. This could be because a higher number of household members requires larger food basket household costs, enough for the larger household size. Household monthly income was also significantly associated with household food (in)security. The more income the household had, the higher the chances it was food-secure. Likewise, if the household had less income, the chances of experiencing food insecurity were higher. Income enabled households to access food they could not grow and to buy the implements they needed to grow indigenous crops. The advantage of having access to purchasing power is that households can also buy quality food to improve their nutritional status.

#### 3.3.2 Factors Associated with Indigenous Crops and Household Food Security

Extended ordered probit regression was used to assess the factors affecting food security among the farming households in study areas. The dependent variable was an ordinal scale that categorised participants through the HFIAS score to determine their household food security status. The dependent variable was coded as 0 = food-secure, 1 = mildly food-insecure, 2 = moderately food-insecure, and 3 = severely food-insecure. Table 5 represents the results. The coefficients of the extended ordered probit model do not entirely represent the magnitude of the effects of the explanatory variables; hence, marginal effects were also computed in Table 6. Multicollinearity was tested for each independent variable via the variance influence factor (VIF). The mean is presented in Table 5.

The findings in Table 5 illustrate that consuming indigenous crops will likely positively influence food security. This means that consuming indigenous crops increases the likelihood of food security among farming households. These results coincide with those of [36,37], who reported that increasing the consumption of various indigenous crops might lead to a stable food security status. The greater number of years a household has been involved in farming increases the likelihood of being food-secure. This means that farming experience is associated with a likelihood of being food-secure. This is confirmed by the marginal effect results in Table 6, which show that farmers with more years of participation in agriculture are likely to fall under food-secure and mildly food-insecure households. This could suggest that farming households with greater experience or exposure have a better understanding and knowledge of the benefits of growing and consuming indigenous crops. Additionally, farming households with years of experience are well informed regarding farmer support programmes, which they likely take advantage of to include indigenous crops in their farming activities. These results coincide with [39,75,76], who reported that farming experience gained through farmer support programs equips farmers with updated agricultural information, which results in better production, yields, and efficient cooperatives that increase the likelihood of household food security.

The findings of this study also show that young men and everyone were perceived as those who should be consuming indigenous crops. This means that young men and everyone will likely be food secure from consuming indigenous crops. While the results suggest that young men and everyone consuming indigenous crops are likely associated with being food secure, the findings of [59,77] clearly stated that the consumption of indigenous crops across age groups is still uncommon, with a limited exception for older people. The current recognition of indigenous crops as perceived in this study may be associated with the perceived medicinal benefits of these crops.

Regarding the marketability of indigenous crops, the findings of this study indicate that sales of indigenous crops are associated with food insecurity. However, the results further showed that if a household happens to sell the indigenous crop in a formal market, they are perceived as likely to be food secure compared to those who would sell in the informal market. This suggests that farming households who select informal and all markets as more suited for these crops are more likely to be food insecure. The perception is not far from South Africa’s realities, where indigenous crops are generally associated with poverty. Therefore, poor households who normally buy from informal markets do not normally consider buying these crops.

On the other hand, when these crops find themselves in formal markets like Wool-worths, where Amadumbe are being sold, the cost is higher and, therefore, is likely generating more money for the farmers. These results also align with [35], where, for example, African leafy vegetables had weak value chain actors, transactions based on spot markets, lack of technical advice on production, and deficiency of contractual agreements between actors. Similarly, Ref. [78] found that limited advances in agronomic advice hinder the improvement of indigenous crops’ market value chains and provide lesser incentives for their continued production and marketing. This reiterates that if indigenous crops were sold in a more formal market, this would increase the incentive to cultivate them among farmers.

The soil analysis information and irrigation systems were associated with food insecurity. This means that assisting farming households with soil analysis and irrigation systems alone may not be enough to help them improve their yield and make a considerable living out of the indigenous crops. Moreover, farming households might not translate the information correctly or purchase the additional nutrients the soil requires. This is in line with results from [79,80], who found that emerging or smallholder farmers require additional assistance during production as they may not have adequate land or proper information to navigate through the different challenges that open-field production has. Furthermore, farmers commonly residing in rural and peri-urban areas also lack tools to sustain well-managed soils and sufficient water supply for domestic needs and irrigation. This may heavily reduce yields, although farmers may have received agricultural support through seeds, garden tools, and fencing [81,82].

Table 6 further shows the analysis of indigenous crop factors that influence household food (in)security; however, it differentiates from Table 5 by showing the odds ratio, standard error and significance values for each category of the dependent value. This allows for a more in-depth analysis of how each independent factor relates to each food (in)security category in the dependent variable. Significant results are further discussed below.

As discussed above, farming households that did not consume indigenous crops were more likely to be food insecure. Table 6 additionally supports that by showing significant values and negative coefficients in the food-secure and mildly food-insecure categories. However, a positive odds ratio value and significant results can be observed in the severely food-insecure category. This means those who do not consume these crops will likely be severely food insecure. This reiterates the importance of these crops in ensuring household food security.

Farming experience increased the likelihood of a household being food secure. This is shown through the positive odds ratio and significant results in the food-secure and mildly food-insecure categories. Mildly food-insecure households likely had slightly less farming experience than food-secure households. On the other hand, the moderately food-insecure and severely food-insecure households had negative odds ratios and significant results. This was expected as farming experience assists farmers in becoming better equipped in their practice and improving their ability to cultivate several crops. Additionally, experience in farming allows farming households to join cooperatives, allowing them to sell their produce in larger markets if combined with other farmers. These results also imply that increased farming experience may increase productivity, thereby contributing to household food security status.

Farming households who perceived older men and everyone as those who should consume indigenous crops were found to be more likely to be food secure or mildly food insecure, as observed through the significant values and positive odds ratio. This suggests that when farming households positively perceive indigenous crops, such as when everyone consumes them, it can improve household food security and possibly their livelihoods. Farming assistance in the form of soil analysis and irrigation equipment increases the likelihood of a household being food insecure. This is shown by the positive odds ratio and significant values in the moderately and severely food-insecure categories. These results also suggest that although farming households require assistance in the form of inputs to be successful in their chosen enterprises, training is also vital for improving overall yields as they may lack the information necessary to produce the required quality and yield within the resources they have or are supplied with. Regarding the marketing results, these results suggest that farmers can be more food secure when selling indigenous crops in formal markets instead of local and informal.

## 4 Conclusions and Recommendations

Improved production and increased variety of indigenous crops cultivated can significantly improve household food security. Greater awareness of these crops is still imperative in South Africa. Indigenous crops are vital because they can adapt to marginal conditions, can contribute to resilient agriculture and sustainable food systems, and are highly nutritious. This study aimed to determine household food security status predictors related to indigenous crop factors among farming households. Gender, household size, and monthly income had a considerable likelihood of contributing to food security. The extended ordered probit model showed that perception, consumption of indigenous crops, farming period, and marketing of indigenous crops significantly influenced household food security. The lack of formal markets for indigenous crops contributes negatively to food security. Low market availability and lack of market knowledge and skills to form, run, or become part of a formal indigenous crop food chain contribute to challenges relating to the sale and purchase of indigenous crops.

This study also concludes that consuming indigenous crops is associated with improved food security status. Farming households not consuming these crops may have severe food insecurity. Generally, male-headed households were found to be more food secure compared to female-headed households. Therefore, female-headed households were more vulnerable to food insecurity. There is a need for targeted programmes to assist and promote female participation in agriculture, particularly in cultivating indigenous crops. To attain the 2030 SDGs, particularly of zero hunger and food security, female- and youth-headed households should be included in the farming-related activities. Government, extension officers, and nutritionists must conduct training workshops to encourage households to grow, market, and buy diversified indigenous crops. Government and policymakers need to include indigenous crops in the national food and nutrition security policy and create formal markets for indigenous crops.

The study results are based on data collected in the KwaZulu-Natal province and may not be generalised as a standard view of all farming households nationally and internationally. There is a need to conduct similar studies across all nine provinces of South Africa. This study focused on food access as part of the elements of food security. Future studies need to include the nutrition element of food security.

## Supplementary Material

Supplementary Materials

## Figures and Tables

**Table 1:** A priori expectations for the explanatory variables used in the models.

Proposed Variable	Type of Measurement	Definition	Hypothesised Effect on HFIAS
Dependant Variable			
Household Food Insecurity Access Scale (Extended Ordered Regression)			
Independent variables			
Indigenous crop access	Categorical	0 = Cultivation from own garden, 1 = Cultivation from cultivated lands from other farmers/gardens, 2 = Collection from wild velds or forests	−
Indigenous crop consumption	Categorical	0 = Yes and 1 = No.	−
Farming period	Categorical	0 = Under 4 years, 1 = 4 to 10 years, 2 = 10 to 20 years, and 3 = Greater than 20 years.	+/−
Indigenous crops’ perception	Categorical	0 = old women; 1 = old men; 2 = young women; 3 = young men; 4 = everyone	+/−
Required farm assistance	Categorical	0 = Seeds, 1 = Garden tools, 2 = Fencing, 3 = Shielding net, and 4 = Soil analysis	−
Indigenous crops’ perceived marketing potential	Categorical	0 = Yes and 1 = No	+/−
Indigenous crops are a suitable marketing channel	Categorical	0 = Local market only, 1 = Informal market only, 2 = Formal market only, and 3 = All markets	+/−

Source: Own analysis.

**Table 2:** Demographic characteristics of farming households.

Variable	Frequency (n)	Percentage (%)	Variable	Frequency(n)	Percentage (%)
Gender			Marital Status		
Male	95	36.5	Married	115	44.2
Female	165	63.5	Unmarried	145	55.8
Age (Years)			Employment Status		
18–24	28	10.8	Unemployed	98	37.7
25–34	43	16.5	Full time	41	15.8
35–44	48	18.5	Part-time	28	10.8
45–54	44	16.9	Informal	5	1.9
55–64	39	15.0	Grant/Pension recipient	85	32.7
65+	58	22.3	Self-employed	3	1.1
Total Household monthly income			Education Level		
R0–R500	48	18.5	None	11	4.2
R501–R1000	48	18.5	Primary	71	27.3
R1001–R1500	45	17.3	Secondary	144	55.4
R1501–R2500	40	15.4	Tertiary	34	13.1
R2501–R3500	25	9.6			
R3501–R4500	30	11.5			
>R4500	24	9.2			

**Table 3:** Percentage response to HFIAS occurrences.

HFIAS Occurrence Questions					
In the Past 30 Days, Did You or Any Member of the Household:	Frequency of Occurrence (%)				
	Yes	No	Rarely (1–2 Times)	Sometimes (3–10 Times)	Often (More Than 10 Times)
Worry about not having enough food	65.8	34.2	47.9	37.4	14.6
Not able to eat the kinds of foods you preferred	77.7	22.3	28.7	40.1	31.2
Have to eat a limited variety of foods	68.1	31.9	46.9	24.3	28.8
Have to eat some foods that you really did not want to eat	76.9	23.1	30.0	42.5	27.5
Have to eat smaller meals than you felt you needed	66.2	33.8	34.9	34.6	12.8
Have to eat fewer meals in a day	70.0	30.0	36.3	49.5	14.3
Ever had no food to eat of any kind in your household?	49.2	50.4	49.2	33.6	17.2
Go to sleep at night hungry	40.4	59.2	26.7	61.9	11.4
Go a whole day and night without eating anything	41.9	58.1	27.5	61.5	5.5

**Table 4:** Household socio-economic profile of four levels of food (in)security.

Demographics	Food-Secure	Mildy Food-Insecure	Moderately Food-Insecure	Severely Food-Insecure	X^2^
Gender					
Male	9	41	28	16	
Female	27	48	66	25	0.057 *
Marital Status					
Married	19	38	41	17	0.904
Unmarried	17	51	53	24	
Number of HH members	36	89	94	41	0.051 *
Employment Status					
Unemployed	15	32	35	16	
Employed	12	34	21	10	0.608
Grant recipient	9	23	38	15	
HH Monthly income					
R0 to R500	5	11	11	5	
R501 to R1000	3	21	34	16	
R1001 to R1500	11	20	28	6	
R1501 to R2500	5	13	7	3	0.059 *
R2501 to R3500	5	4	4	4	
R3501 to R4500	2	7	7	2	
>R4500	5	13	3	5	
Educational Level					
None	5	9	15	7	
Primary	9	22	20	10	0.525
Secondary	15	50	57	22	
Tertiary	7	13	8	6	

* = significant at the 0.1 level.

**Table 5:** Factors associated with indigenous crops and household food security.

Variables	Coefficient	S. E	*p*>z
Indigenous crop access			
Collection from own garden	(Base)		
Collection from cultivated lands	−0.027	0.249	0.913
Collection from wild veld or forest	−0.388	0.283	0.170
Indigenous crop consumption			
Yes	(Base)		
No	1.174	0.468	0.012 **
Farming Period			
Under 4 years			
4 to 10 years	−1.015	0.293	0.001 ***
10 to 20 years	−1.117	0.318	0.000 ***
Greater than 20 years	−1.164	0.332	0.000 ***
Indigenous crops’ perception			
Old women	(Base)		
Old men	−0.006	0.278	0.983
Young men	−1.344	0.454	0.003 ***
Everyone	−1.083	0.237	0.000 ***
Indigenous crops’ marketing potential			
Yes	(Base)		
No	−0.400	0.183	0.029 **
Perceived suitable marketing channel			
Local market only	(Base)		
Informal market	0.845	0.435	0.052 *
Formal market	−0.956	0.363	0.008 ***
All markets	1.041	0.211	0.000 ***
Required Assistance			
Seeds	(Base)		
Garden tools	0.091	0.354	0.796
Fencing	-0.286	0.254	0.260
Soil analysis	1.060	0.404	0.009 ***
Irrigation	1.423	0.688	0.038 **
HFIAS CATEGORIES			
cut1	−2.988	0.436	
cut2	−1.223	0.409	
cut3	0.199	0.390	
Log-likelihood	−192.724		
Wald chi2	83.180		
Prob > Chi2	0.000 ***		
Akaike’s information criterion	425.443		
Bayesian information criterion	490.598		
Mean VIF	1.88		

* = significant at the 0.1 level;

** = significant at the 0.05 level

*** = significant at the 0.01 level.

**Table 6:** Extended ordered probit analysis marginal effects of factors associated with indigenous crops and household food security.

Categorical	Variables	Food Secure			Mildly Food Insecure			Moderately Food Insecure			Severely Food Insecure		
		Odds Ratio	Standard Error	*p*>z	Odds Ratio	Standard Error	*p*>z	Odds Ratio	Standard Error	*p*>z	Odds Ration	Standard Error	*p*>z
	Own Garden	Base											
Indigenous Crop Access	Cultivated Lands	0.003	0.031	0.913	0.004	0.041	0.913	–0.003	0.027	0.913	–0.005	0.045	0.914
	Collection from wild velds or forests	0.057	0.043	0.189	0.055	0.038	0.148	–0.051	0.037	0.171	–0.061	0.044	0.169
Consumption of Indigenous Crops	Yes	Base											
	No	–0.094	0.024	0.000 ***	–0.217	0.082	0.008 ***	0.047	0.032	0.140	0.265	0.125	0.034 **
	Under 4 years	Base											
Farming Period	4 to 10 years	0.088	0.024	0.000 ***	0.190	0.056	0.001 ***	–0.053	0.022	0.015 **	–0.225	0.073	0.002 ***
	10 to 20 years	0.103	0.031	0.001 ***	0.204	0.058	0.000 ***	–0.067	0.028	0.019 **	–0.241	0.075	0.001 ***
	>20 years	0.111	0.035	0.002 ***	0.210	0.059	0.000 ***	–0.073	0.030	0.014 **	–0.247	0.076	0.001 ***
Perception of Indigenous Crops	Old women	Base											
	Old men	0.000	0.018	0.983	0.001	0.064	0.983	0.000	0.013	0.983	–0.001	0.069	0.983
	Young men	0.197	0.096	0.041 **	0.216	0.049	0.000 ***	–0.221	0.087	0.011 **	–0.192	0.052	0.000 ***
	Everyone	0.139	0.034	0.000 ***	0.201	0.048	0.000 ***	–0.166	0.039	0.000 ***	–0.175	0.046	0.000 ***
Required Assistance	Seeds	Base											
	Garden Tools	–0.012	0.046	0.789	–0.015	0.059	0.804	0.012	0.045	0.791	0.015	0.060	0.802
	Fencing	0.046	0.044	0.295	0.037	0.030	0.212	–0.043	0.040	0.282	–0.040	0.033	0.228
	Soil Analysis	–0.084	0.023	0.000 ***	–0.211	0.081	0.009 ***	0.059	0.236	0.012 **	0.236	0.107	0.028 **
	Irrigation	–0.093	0.023	0.000 ***	–0.279	0.119	0.019 **	0.032	0.074	0.666	0.340	0.200	0.089 *
Indigenous crops’ perceived marketing potential	Yes	Base											
	No	0.052	0.023	0.026 **	0.067	0.034	0.047 **	–0.050	0.024	0.041 **	–0.069	0.032	0.033 **
Indigenous crops’ perceived marketing channels	Local Market Only	Base											
	Informal Market Only	–0.117	0.046	0.010 **	–0.131	0.090	0.146	0.114	0.047	0.015 **	0.134	0.086 *	0.120
	Formal Market Only	0.244	0.100	0.014 **	–0.031	0.047	0.511	–0.152	0.049	0.002 ***	–0.061	0.021 **	0.004 ***
	All market	–0.132	0.032	0.000 ***	–0.173	0.038	0.000 ***	0.126	0.031	0.000 ***	0.179	0.037 **	0.000 ***

Note: Dy/dx (odds ratio) represents the discrete change of variable from 0 to 1 (or 2…). HFIAS (Y) is the dependent variable and is represented by four levels, starting from food secure ascending to severely foodr insecure. *, **, ***: significant at 10%, 5%, and 1%.

## Data Availability

The data are not publicly available due to the protection of information of the participants.
