# Supplementary material for: The Association of Socio-Economic Factors and Indigenous Crops on the Food Security Status of Farming Households in KwaZulu-Natal Province
Source: Agriculture (Basel). Author manuscript; Available in PMC 2024 Jul 29. (PMC7616313; doi:10.3390/agriculture14030415)
Supplement: Supplementary Materials [file EMS197330-supplement-Supplementary_Materials.zip › agriculture-2846553-supplementary.pdf]

Supplementary Material S1

Household Food Insecurity Access Scale (HFIAS) Tool

Questions		Reply
Q1	In the past four weeks, did you worry that your household would not have enough food?	0 = No; 1 = Yes
Q1a	How often did this happen?	1 = Rarely
Q2	In the past four weeks, were you or any household member not able to eat the kinds of foods you preferred because of a lack of resources?	1= Rarely
Q2a	How often did this happen?	1 = Rarely
		2 = Sometimes
		3 = Often
Q3	In the past four weeks, did you or any household member have to eat a limited variety of foods due to a lack of resources?	0 = No; 1 = Yes
Q3a	How often did this happen?	1 = Rarely
		2 = Sometimes
		3 = Often
Q4	In the past four weeks, did you or any household member have to eat some foods that you really did not want to eat because of a lack of resources to obtain other types of food?	0 = No; 1 = Yes
Q4a	How often did this happen?	1 = Rarely
		2 = Sometimes
		3 = Often
Q5	In the past four weeks, did you or any household member have to eat a smaller meal than you felt you needed because there was not enough food?	0 = No; 1 = Yes
Q5a	How often did this happen?	1 = Rarely
		2 = Sometimes
		3 = Often
Q6	In the past four weeks, did you or any household member have to eat fewer meals in a day because there was not enough food?	0 = No; 1 = Yes
Q6a	How often did this happen?	1 = Rarely
		2 = Sometimes
		3 = Often

Q7	In the past four weeks, was there ever no food to eat of any kind in your household because of lack of resources to get food?	0 = No; 1 = Yes
Q7a	How often did this happen?	1 = Rarely
		2 = Sometimes
		3 = Often
Q8	In the past four weeks, did you or any household member go to sleep at night hungry because there was not enough food?	0 = No; 1 = Yes
Q8a	How often did this happen?	1 = Rarely
		2 = Sometimes
		3 = Often
Q9	In the past four weeks, did you or any household member go a whole day and night without eating anything because there was not enough food?	0 = No; 1 = Yes
Q9a	How often did this happen?	1 = Rarely
		2 = Sometimes
		3 = Often
Note: Questions 1 to 9 are the **occurrence questions** while questions 1a to 9a are the **frequency-of-occurrence questions**.		
